# Pyomelanin Formation in *Aspergillus fumigatus* Requires HmgX and the Transcriptional Activator HmgR but Is Dispensable for Virulence

**DOI:** 10.1371/journal.pone.0026604

**Published:** 2011-10-27

**Authors:** Sophia Keller, Juliane Macheleidt, Kirstin Scherlach, Jeannette Schmaler-Ripcke, Ilse D. Jacobsen, Thorsten Heinekamp, Axel A. Brakhage

**Affiliations:** 1 Department of Molecular and Applied Microbiology, Leibniz Institute for Natural Product Research and Infection Biology, Hans Knöll Institute (HKI), Jena, Germany; 2 Department of Biomolecular Chemistry, Leibniz Institute for Natural Product Research and Infection Biology, Hans Knöll Institute (HKI), Jena, Germany; 3 Department of Microbial Pathogenicity Mechanisms, Leibniz Institute for Natural Product Research and Infection Biology, Hans Knöll Institute (HKI), Jena, Germany; 4 Department of Microbiology and Molecular Biology, Friedrich Schiller University, Jena, Germany; New Jersey Medical School, University of Medicine and Dentistry of New Jersey, United States of America

## Abstract

The opportunistic human pathogenic fungus *Aspergillus fumigatus* is able to produce the dark brown pigment pyomelanin by degradation of L-tyrosine. Pyomelanin was shown to protect the fungus against reactive oxygen intermediates as well as cell wall disturbing compounds and is therefore assumed to protect against immune effector cells during the infection process. Several genes for tyrosine degradation and pyomelanin formation are organized in a cluster in the genome of *A. fumigatus*. Here, we aimed at further analyzing tyrosine degradation and a possible role of pyomelanin in virulence. For this purpose, the function of two not yet characterized genes of the cluster, i.e., *hmgX* and *hmgR*, was analyzed. Generation of corresponding gene deletion mutants and reconstituted strains revealed that *hmgX* and *hmgR* are essential for tyrosine degradation. Both mutants, Δ*hmgX* and Δ*hmgR*, were not able to use tyrosine as sole carbon or nitrogen source and revealed impaired pyomelanin production. HmgR harbors a Zn(II)2Cys6-DNA binding domain. Analyses of the steady state mRNA levels revealed that HmgR acts as a transcriptional activator for the genes of the tyrosine degradation cluster. Consistently, an HmgR-eGFP fusion protein was localized in the nucleus of *A. fumigatus* cells. By contrast, HmgX was found to be localized in the cytoplasm and does not contribute to regulation of gene transcription. HPLC analyses showed that HmgX is crucial for the conversion of *p*-hydroxyphenylpyruvate to homogentisic acid, the main intermediate in pyomelanin formation. Thus, HmgX is supposed to function as an accessory factor to mediate specific activity of HppD. Remarkably, the ability to degrade tyrosine and to form pyomelanin is dispensable for virulence of *A. fumigatus* in a murine infection model.

## Introduction

The ascomycete fungus *Aspergillus fumigatus* is the clinically most important member of the genus *Aspergillus* [1]. *A. fumigatus* is a ubiquitous soil inhabitant feeding on organic material, thereby playing a key role in recycling carbon and nitrogen sources [2]. *A. fumigatus* produces small conidia that are distributed in the air and are continuously inhaled by breathing organisms [3]. Normally, inhaled conidia are cleared by the innate immune system [4]. Patients with a compromised immune system are not able to clear inhaled spores and therefore are at high risk to acquire an invasive infection.

*A. fumigatus* produces the pigment dihydroxynaphthalene (DHN) melanin, responsible for the characteristic gray-green color of the conidia. In general, melanins play protective roles in fungi and other organisms. For example, they protect against UV radiation, enhance cell wall integrity and mediate increased resistance against enzymatic lysis, oxidative agents, and extreme temperature [5]. In some plant and animal pathogenic fungi, the protective and stabilizing activities of melanins represent virulence determinants [6], [7]. Melanins reduce the susceptibility against reactive nitrogen and oxygen intermediates (ROI) produced by the host immune system [8]. In *A. fumigatus*, the polyketide synthase PksP is the key enzyme in the biosynthesis of DHN-melanin. Mutants deficient for PksP produce white conidia and are attenuated in virulence [9]. DHN-melanin has been shown to protect *A. fumigatus* against ROI, derived from host immune effector cells. It also has an effect on phagolysosome maturation and thereby killing of *A. fumigatus* conidia [6], [10], [11], [12].

**Figure 1:**
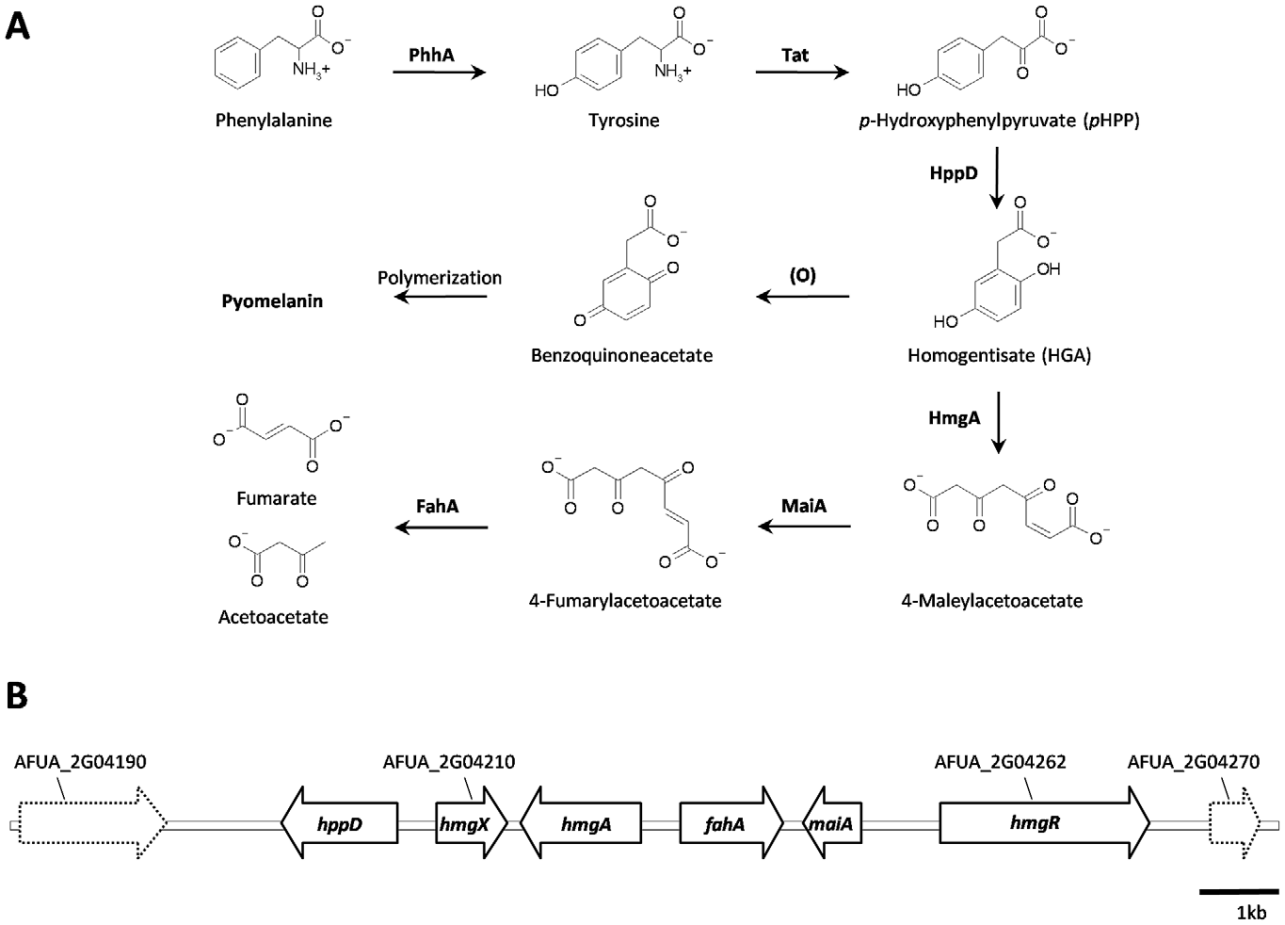
Tyrosine degradation pathway and cluster organization. (A) Enzymes involved in degradation of L-phenylalanine and L-tyrosine are phenylalanine hydroxylase (PhhA), tyrosine aminotransferase (Tat), *p*-hydroxyphenylpyruvate dioxygenase (HppD), homogentisate dioxygenase (HmgA), 4-maleylacetoacetate isomerase (MaiA) and 4-fumarylacetoacetate hydrolase (FahA). Oxidation (O) and polymerization of homogentisate leads to formation of pyomelanin. (B) The tyrosine degradation cluster in *A*. *fumigatus* [13] encodes four enzymes involved in degradation of tyrosine as well as HmgX (AFUA_2G04210) and the transcriptional regulator HmgR (AFUA_2G04262). Genes are indicated as arrows.

Recently, it was shown that *A. fumigatus* is able to produce the brownish pigment pyomelanin as an alternative melanin [13]. At first, pyomelanin was identified in the bacterium *Pseudomonas aeruginosa* [14], and later on it was also described for other bacteria and fungi, e.g., *Shewanella colwelliana* [15], *Yarrowia lipolytica* [16] and *Vibrio cholerae* [17]. Pyomelanin is produced via degradation of L-tyrosine with homogentisic acid (HGA) as the main intermediate (Figure 1A). In higher eukaryotes, especially in humans, the HGA pathway has been subject to detailed investigations as the origin of several metabolic disorders, e.g., phenylketonuria, alkaptonuria, tyrosinaemia, and Hawkinsinuria, that have been linked to enzymatic defects in phenylalanine and tyrosine catabolism [18]. The tyrosine degradation pathway was also investigated in the model organism *Aspergillus nidulans* [19], [20]. In *A. fumigatus*, two enzymes of the tyrosine degradation pathway, *p*-hydroxyphenylpyruvate dioxygenase (HppD) and homogentisate dioxygenase (HmgA), were recently characterized in detail [13]. Deletion of *hppD* prevents synthesis of HGA and consequently pyomelanin. By contrast, deletion of *hmgA* prevents the enzymatic degradation of HGA, resulting in HGA accumulation and increased pyomelanin formation.

Remarkably, genes involved in tyrosine degradation, *hppD*, *hmgA*, *maiA* and *fahA*, are organized in a cluster in the genome of *A. fumigatus*. Here, we present data on the functional characterization of two previously uncharacterized genes, AFUA_2G04210 and AFUA_2G04262, that are also localized within the tyrosine degradation cluster in *A. fumigatus* (Fig. 1B). We were able to show that both genes play crucial roles in tyrosine degradation and pyomelanin formation. However, the ability to degrade tyrosine and to form pyomelanin does not contribute to fungal induced mortality at least in a murine infection model for invasive pulmonary aspergillosis.

## Materials and Methods

### Fungal strains and growth conditions

All *A. fumigatus* strains used in this study are listed in Table S1. Wild-type strains CEA10 and Δ*akuB* [21] were used for generation of mutants. *A. fumigatus* was cultivated at 37°C on *Aspergillus* minimal medium (AMM) agar plates or in AMM as described previously [22]. Unless noted otherwise, 50 mM glucose and 70 mM nitrate were used as sole carbon and nitrogen sources. The medium was supplemented with 0.1 mg/l pyrithiamine (Sigma-Aldrich, Germany) or 250 mg/l hygromycin (InvivoGen, France) when required. Conidia were harvested in sterile water. For protein extraction, 1×10^7^ conidia were cultivated in 100 ml AMM for 16 h. When indicated, L-tyrosine was added to the medium in a final concentration of 10 mM. After further cultivation for 12 h the mycelium was harvested using miracloth (Calbiochem, Germany).

### Manipulation of DNA, Southern blot and Northern blot analyses

Manipulation of DNA was carried out according to standard procedures [23]. Sequence information were obtained from the Central *Aspergillus* Data REpository C*A*DRE (http://www.cadre-genomes.org.uk) [24]. All oligonucleotides used in this study are listed in Table S2. Chromosomal DNA of *A. fumigatus* was isolated using the MasterPure Yeast DNA purification kit (Epicentre Biotechnologies, USA). Southern blot analysis was performed as described previously [25]. For RNA isolation, 5×10^7^ conidia of *A. fumigatus* were pre-cultivated in 50 ml AMM for 16 h. Then, either supplements (L-phenylalanine, L-tyrosine, *p*-hydroxyphenylpyruvate or homogentisate) were added in a final concentration of 10 mM or the mycelium was shifted to AMM without a carbon or nitrogen source and harvested after additional incubation for up to 12 h. Total RNA was isolated using the TriSure reagent (Bioline, Germany) according to the manufacturer's instructions. For Northern blot analyses, 10 µg of RNA were separated on a denaturing agarose gel and transferred onto a Hybond N+membrane (GE Healthcare Bio-Sciences, Germany). Probe labeling, hybridization and detection were performed with the digoxigenin (DIG) labeling mix, DIG Easy Hyb, and the CDP-Star ready-to-use kit (Roche, Germany) according to the manufacturer's recommendations. cDNA synthesis and reverse transcription PCR was performed as described previously [13].

### Construction of recombinant *A. fumigatus* strains

To generate an *hmgX* deletion strain, a DNA fragment was created by PCR-based amplification of a 1.5 kb sequence containing the *hmgX* gene and flanking regions using primers hmgX5for and hmgX3rev and wild-type genomic DNA as template. The resulting PCR product was cloned into pCR2.1 (Invitrogen, Germany), yielding pCR2.1 *hmgX*. The pyrithiamine resistance cassette (*ptrA*) was obtained from plasmid pCR2.1 Mpka/ptrA (kindly provided by V. Valiante, HKI Jena, Germany) by restriction with *Dra*I and *Sma*I. The *ptrA* fragment was ligated into pCR2.1 *hmgX* at a single *Hinc*II site, located in the center of the *hmgX* gene *via* blunt end cloning, resulting in vector pCR2.1 *hmgX*-*ptrA*. Using this plasmid as template, a 3.7 kb DNA fragment was amplified by PCR using Phusion high fidelity polymerase (Finnzymes, Finland) with primers hmgX5for and hmgX3rev and used for transformation of *A. fumigatus*.

Complementation of strain Δ*hmgX* was achieved by transformation of strain Δ*hmgX* with plasmid pCR2.1 *hmgX*. The ability to grow on tyrosine as sole carbon source was used as selective marker and the generated strain was designated as *hmgX*c.

For deletion of both *hmgX* and the adjacent *hmgA* gene, flanking regions were generated in separate PCR reactions using primer pairs hmgX5for/hmgX-ptrA5rev and hmgA5rev/hmgA-ptrA5for. The *ptrA* fragment was amplified from plasmid pSK275 [26] with primers ptrAforII and ptrArevII. All three DNA fragments were subjected to a fusion PCR using primers hmgX5for and hmgA5for, resulting in amplification of a 3.5 kb construct that was used to transform *A. fumigatus* strain Δ*akuB*.

Generation of an *hmgX*-*egfp* fusion strain was done as follows: Using primers HmgX-Acc65Ifor and HmgX-BamHIrev the *hmgX* gene and promoter region were amplified with genomic wild-type DNA as template. The PCR product encoding *Bam*HI and *Acc*65I cleavage sites at its ends was cloned into pJET1.2 (Fermentas, Germany) yielding plasmid pJET-*hmgX*. Plasmids pJET-*hmgX* and pUCGH [27] were then digested using restriction endonucleases *Acc*65I and *Bam*HI, thereby removing the *otef* promoter sequence present on plasmid pUCGH. The *hmgX* fragment was finally ligated to pUCGH vector backbone *via Bam*HI and *Acc*65I sites resulting in plasmid pUCGH*hmgX*p-*hmgX* that was used to transform *A. fumigatus* wild-type strain CEA10.

To obtain the *hmgR* deletion plasmid, the *hmgR* gene, including up- and downstream flanking regions of 1.0 kb, was amplified by PCR using the oligonucleotides Tf_Tyr_up and Tf_Tyr_down. The generated DNA fragment was cloned into plasmid pCR2.1. The obtained plasmid pCR2.1 *hmgR* was used as template for an inverse PCR with the primers Tf_Tyr_SfiI_up and Tf_Tyr_SfiI_down to modify the ends of the flanking regions with *Sfi*I restriction sites and to remove the *hmgR* coding sequence. After *Sfi*I digestion of the PCR product, the pyrithiamine resistance gene (*ptrA*) from plasmid pSK275 was integrated into the *Sfi*I restriction sites, resulting in the deletion plasmid pCR2.1Δ*hmgR-ptrA.* For transformation of *A. fumigatus,* the *ptrA* gene with the *hmgR* flanking regions was amplified by PCR with the oligonucleotides Tf_Tyr_up and Tf_Tyr_down.

The phenotype of the Δ*hmgR* deletion mutant was reconstituted with the help of plasmid pUCGH-*hmgR*, which possesses the fusion gene *hmgR*-*egfp* under the control of the native *hmgR* promoter. To obtain pUCGH-*hmgR*, the *hmgR* gene with its 1.0 kb promoter region was amplified by PCR using the oligonucleotides PyoTf_BamHIrev and PyoTf5′_Acc65I. The generated PCR product with introduced *Bam*HI and *Acc*65I restriction sites was then cloned into plasmid pJET1.2, yielding pJET-*hmgR*. The DNA fragment was inserted into pUCGH *via* the *Acc*65I and *Bam*HI restriction sites. The resulting plasmid pUCGH-*hmgR* was used to transform *A. fumigatus* strain Δ*hmgR*.

### Extraction of proteins from *A. fumigatus* and enzyme assays

Activity of the enzymes tyrosine aminotransferase (Tat) and homogentisate dioxygenase (HmgA), as well as formation of HGA from *p*HPP, was determined for protein crude extracts of different *A. fumigatus* strains. Protein extracts were obtained by sonification in 50 mM potassium phosphate buffer (pH 7.0) followed by centrifugation for 15 min at 4°C and 16,000×g. The supernatant was used as protein crude extract in enzyme assays as described below. Determination of protein concentration was performed using Coomassie Plus Protein Assay (Pierce Biotechnology, USA).

For determination of Tat activity the method of Collier and Kohlhaw [28] was applied, with slight modifications. In brief, 100 mM potassium phosphate buffer (pH 7.5), 0.2 mM pyridoxal phosphate, 2 mM L-tyrosine, 100 µ/ml protein crude extract and 1 µM sulcotrione (Sigma-Aldrich, Germany) were pre-incubated at 37°C for 15 min. To start the reaction 20 mM α-ketoglutarate was added. The final reaction volume was 500 µl. After 10 min at 37°C the reaction was stopped with 500 µl 2 M NaOH thus leading to the conversion of *p*HPP to *p*-hydroxybenzaldehyde [29], which was measured spectrophotometrically at 330 nm. To determine the specific activity of Tat the molar extinction coefficient 19,500 M^−1^ cm^−1^ was employed [28].

Activity of HmgA was measured using the method described by Fernandez-Canon and Penalva [30] modified according to Schmaler-Ripcke, *et al.* [13].

### HHGA formation by protein extracts

To determine activity of HppD, conversion of *p*HPP to HGA by protein crude extracts was measured by HPLC. Therefore, the HmgA activity assay was adapted as follows: 50 mM potassium phosphate buffer (pH 7.0), 2 mM ascorbate, 50 µM FeSO_4_ and 100 µg/ml protein extract were mixed and pre-incubated for 15 min at room temperature. After addition of 200 µM *p*HPP in a final volume of 200 µl the enzymatic reaction proceeded at room temperature for 10 min and was stopped with 50 µl 10%(w/v) trichloroacetic acid. Proteins were precipitated by centrifugation for 10 min at 16,000×g and 4°C. The supernatant was analyzed by HPLC.

### HPLC analysis

To monitor formation of *p*HPP and HGA by *A. fumigatus*, samples of culture supernatants were collected at different time points during cultivation in presence of tyrosine and analyzed by HPLC as described by Schmaler-Ripcke, *et al.* [13].

### Fluorescence and light microscopy

For microscopic analysis, *A. fumigatus* strains were cultivated over night on coverslips with or without tyrosine. For staining of nuclei, Hoechst 33342 (Invitrogen, Germany) was added to the medium in a final concentration of 10 µg/ml. Microscopic photographs were taken on a Leica DM4500B digital fluorescence microscope and for documentation a Leica DFC480 digital camera (Leica Microsystems, Germany) was used. Images were obtained and processed with Leica Application Suite 2.5.0R1.

### Animal infection model

The virulence of the *A. fumigatus* mutant Δ*hppD* and the corresponding complemented strain *hppD*c was tested in an established murine model for invasive pulmonary aspergillosis [31], [32], [33]. In brief, female BALB/c or CD-1 mice were immunosuppressed with cortisone acetate (25 mg/mouse intraperitoneally; Sigma-Aldrich, Germany) on days−3 and 0. Mice were anesthetized and intranasally infected with 25 µl of a fresh suspension containing 1×10^5^ conidia. A control group was mock-infected with PBS to monitor the influence of the immunosuppression. The health status was monitored at least twice daily for 14 days and moribund animals (defined by severe dyspnoea and/or severe lethargy) were sacrificed. Infections were performed with a group of 10 mice for each tested strain. Lungs from euthanized animals were removed, and either stored in RNA*later* (Qiagen, Germany) for RNA extraction or fixed in formalin and paraffin-embedded for histopathological analyses according to standard protocols. RNA isolation and first-strand cDNA synthesis from infected lungs was performed as described previously [13], [34].

### Ethics statement

Mice were cared for in accordance with the principles outlined by the European Convention for the Protection of Vertebrate Animals Used for Experimental and Other Scientific Purposes (European Treaty Series, no. 123; http://conventions.coe.int/Treaty/en/Treaties/Html/123.htm). All animal experiments were in compliance with the German animal protection law and were approved by the responsible Federal State authority “Thüringer Landesamt für Lebensmittelsicherheit und Verbraucherschutz” and ethics committee “Beratende Komission nach § 15 Abs. 1 Tierschutzgesetz” with the permit number 03-001/08.

## Results

### Tyrosine degradation pathway and cluster organization

Several genes involved in tyrosine catabolism are organized in a cluster within the genome of *A. fumigatus*. As shown in Fig. 1B, two genes, AFUA_2G04262 and AFUA_2G04210, are part of the cluster, whereas their function has not been analyzed before. AFUA_2G04262 encodes a putative C6 zinc finger transcription factor and is in the following named *hmgR*. The predicted transcript of 2247 bp is composed of four exons and the deduced protein has a size of 748 amino acids. AFUA_2G04210, in the following designated *hmgX*, has a transcript length of 771 bp, and does not contain any intron. No conserved domains and no similarities to any proteins with known function can be attributed to HmgX.

To investigate whether HmgX is also present in other organisms, BLAST analysis with the *A. fumigatus* HmgX amino acid sequence was performed using the tblastn algorithm (www.ncbi.nlm.nih.gov/BLAST). Highest identities were found within *Ascomycota*, all predicted as hypothetical proteins. In most ascomycetes that were sequenced up to date, the *hppD* gene is located adjacent to *hmgX*. In many cases, as it is found in *A. fumigatus,* both genes appear to be under control of a bi-directional promoter. In other organisms, such as *Homo sapiens*, the nematode *Caenorhabditis elegans* and the slime mold *Dictyostelium discoideum*, no homologs of *hmgX* were found.

Alignment of the *A. fumigatus* HppD amino acid sequence to the sequence of three ascomycetes that encode an HmgX homolog (*Coccidioides immitis*, *Neurospora crassa*, *Magnaporthe grisea*) and three species without any apparent HmgX homolog (*H. sapiens*, *C. elegans*, *D. discoideum*) (www.ebi.ac.uk/Tools/clustalw2) revealed strong conservation of the HppD sequence among all organisms, especially with regard to the C-terminus. However, at the N-terminus a 13 amino acid sequence is specific for ascomycete fungi and missing in other organisms which do not have an HmgX homolog (Fig. S1).

### Transcriptional analysis of *hmgR* and *hmgX* and phenotypic characterization of *hmgX* and *hmgR* mutant strains

**Figure 2:**
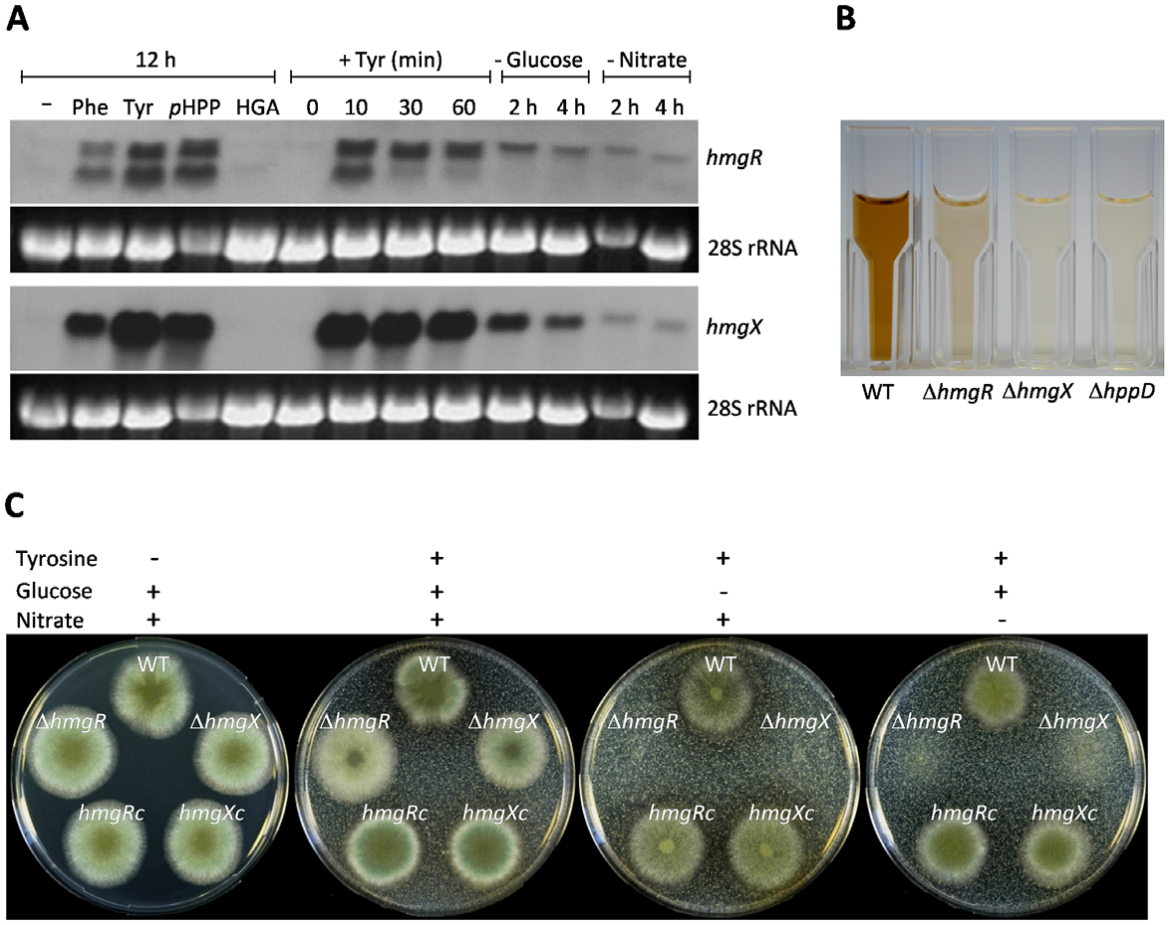
Transcriptional analysis of *hmgR* and *hmgX* and phenotypic characterization of *hmgX* and *hmgR* mutant strains. (A) *A. fumigatus* wild type was pre-cultivated in AMM and Northern blot analyses were performed to determine mRNA steady state levels of *hmgR* and *hmgX* in cultures with (+) or without (-) addition of L-phenylalanine (Phe), L-tyrosine (Tyr), *p*-hydroxyphenylpyruvate (*p*HPP) or homogentisate (HGA). A time course of induction of transcription of *hmgR* and *hmgX* by addition of tyrosine was analyzed in *A. fumigatus* wild type pre-cultivated for 16 h in AMM at the indicated time points. Transcript levels for *hmgR* and *hmgX* were also determined in cultures starved in glucose (-Glucose) or nitrate (-Nitrate). 28S rRNA bands are shown as loading control. (B) Formation of pyomelanin in the wild type (WT) and Δ*hmgR*, Δ*hmgX* and Δ*hppD* mutants cultivated for 64 h in AMM containing tyrosine. (C) Growth of *A. fumigatus* wild type (WT), Δ*hmgX* and Δ*hmgR* mutants and reconstituted strains *hmgR*c and *hmgX*c on different minimal medium agar plates.

**Figure 3:**
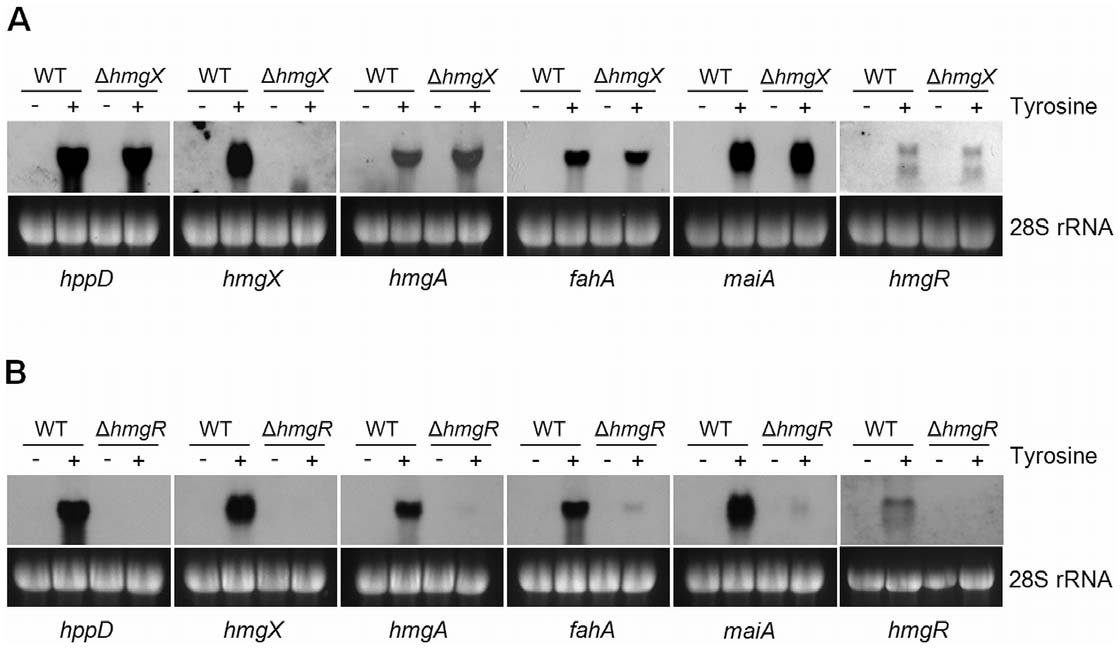
Northern blot analysis of genes of the tyrosine degradation cluster in Δ*hmgR* and Δ*hmgX* mutants. mRNA steady state levels of genes of the tyrosine degradation cluster in the wild type and in strain Δ*hmgX* (A) and strain Δ*hmgR* (B) were determined in cultures with (+) or without (-) addition of tyrosine. 28S rRNA bands are shown as loading control.

By Northern blot analysis we were able to show that transcription of all genes of the cluster is induced in the presence of tyrosine (Fig. 2A and Fig. 3). By contrast, transcription of the adjacent genes AFUA_2G04270 and AFUA_2G04190 was not influenced by tyrosine (Fig. S2). To further analyze conditions resulting in transcriptional activation of genes of the tyrosine degradation cluster, the mRNA steady state level of *hmgR* and *hmgX* was determined after addition of phenylalanine, tyrosine, *p*HPP, and HGA, as well as under glucose or nitrogen starvation conditions. Both, phenylalanine and *p*HPP induced transcription of *hmgR* and *hmgX* to a slightly lesser extent compared to induction by tyrosine. Interestingly, for *hmgR* two transcripts of different length were detected. It remains to be elucidated, whether these two transcripts are the result of alternative splicing events or indicate the use of alternative transcription start points. Glucose and nitrogen starvation also weakly induced transcription of *hmgX* and *hmgR* (Fig. 2A).

To functionally characterize HmgX and HmgR, the mutant strains Δ*hmgX* and Δ*hmgR*, as well as the corresponding reconstituted strains *hmgX*c and *hmgR*c were generated (Fig. S3, S4). Phenotypical analysis of Δ*hmgX* and Δ*hmgR* confirmed a key role for HmgX and HmgR in pyomelanin formation and tyrosine degradation: When cultivated in AMM in the presence of tyrosine, Δ*hmgX* was unable to produce pyomelanin, similar to the Δ*hppD* mutant, and Δ*hmgR* exhibited a drastically reduced pigment production in comparison to the wild type (Fig. 2B). *A. fumigatus* is able to use tyrosine as carbon and nitrogen source. Therefore, the Δ*hmgX* and Δ*hmgR* mutants were analyzed with regard to their ability to use tyrosine as sole carbon or nitrogen source. Both mutants grew normally with glucose and nitrate as sole carbon and nitrogen sources (Fig. 2C). However, Δ*hmgX* and Δ*hmgR* were unable to grow on solid media with tyrosine as sole carbon or nitrogen source in contrast to the wild type and the complemented strains *hmgX*c and *hmgR*c. On agar plates containing tyrosine, the wild type and reconstituted strains cleared the tyrosine crystals in the medium and produced pyomelanin, visible as a halo surrounding the colonies. This is in contrast to Δ*hmgX* and Δ*hmgR* in which neither tyrosine degradation nor pyomelanin formation occurred.

To test whether HmgX or HmgR act as transcriptional regulator of tyrosine catabolism, the mRNA steady state level of all genes within the cluster was determined in the wild type and the Δ*hmgX* and Δ*hmgR* mutants (Fig. 3). While transcript levels of *hmgR, hmgA*, *fahA*, *hppD*, and *maiA* were not affected in the *hmgX* mutant, transcription of all cluster genes was nearly abolished in the Δ*hmgR* mutant.

### Localization of HmgR-eGFP and HmgX-eGFP fusion proteins

**Figure 4:**
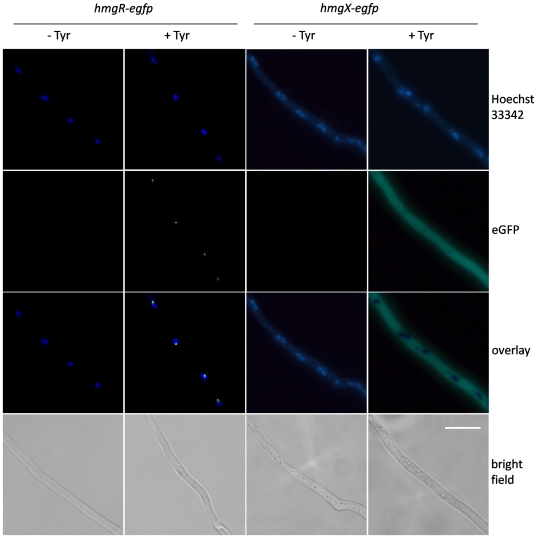
Localization of HmgR-eGFP and HmgX-eGFP fusion proteins. For localization of HmgR and HmgX, conidia of strains *hmgR-egfp* and *hmgX-egfp* were grown on coverslips in AMM with (+) or without (-) addition of tyrosine. Transcription of both *egfp*-fusion constructs is under control of the native *hmgR* or *hmgX* promoter. Nuclei were stained with Hoechst 33342. Bright field, nuclei staining, eGFP-fluorescence and the resulting overlay of the fluorescence images are shown for both strains in dependency of tyrosine. Scale bar = 10 µm.

To determine the localization of HmgR within *A. fumigatus* cells, a mutant strain expressing an HmgR-eGFP fusion protein in the Δ*hmgR* background was generated (Fig. S5). Transcription of *hmgR*-*egfp* was controlled by the native *hmgR* promoter. Transformants regained the ability to produce pyomelanin and to use tyrosine as sole carbon and nitrogen source, confirming functionality of the HmgR-eGFP-fusion protein. Using fluorescence microscopy, localization of HmgR-eGFP was determined (Fig. 4, left panel). Conidia were cultivated on coverslips in AMM with or without addition of tyrosine. Without tyrosine, no fluorescence in the fungal cells was visible. However, in the presence of tyrosine, HmgR-eGFP was apparent in the nuclei. Fluorescence analysis of an *A. fumigatus* mutant constitutively producing HmgR-eGFP (*otefp*-*hmgR*-*egfp*, data not shown) revealed perpetual nuclear localization of HmgR, suggesting that transport of HmgR to the nucleus occurs independently from the presence of tyrosine. In contrast to nuclear HmgR, an HmgX-eGFP fusion protein was found to be present exclusively in the cytoplasm (Fig. 4, right panel). Transcriptional control of *hmgX*-*egfp* was mediated by the native *hmgX* promoter and consequently fluorescence beyond the background level only occurred in the presence of tyrosine.

### Determination of Tat and HmgA enzyme activities in the Δ*hmgX* mutant and analysis of *p*HPP and HGA production

**Figure 5:**
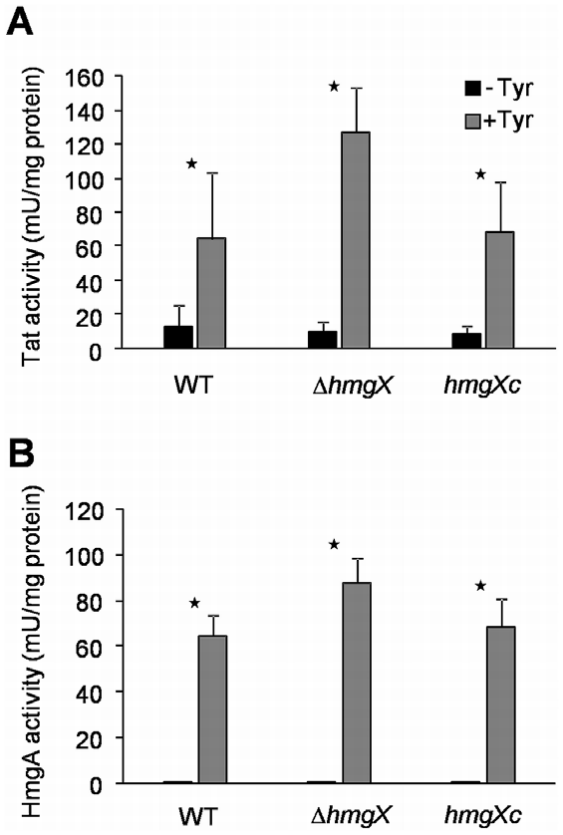
Determination of specific enzyme activity. The wild type and strains Δ*hmgX* and *hmgX*c were cultivated in AMM with or without addition of tyrosine. Protein crude extracts were used in enzyme assays to determine specific activity of tyrosine aminotransferase (A) and homogentisate dioxygenase (B). Mean values and standard deviations were calculated from three independent experiments. Significance was calculated by a Student's *t*-test and an asterisk indicates *P* values<0.05.

The role of HmgX in tyrosine degradation was further investigated by enzyme assays. Specific activity of tyrosine aminotransferase (Tat) and homogentisate dioxygenase (HmgA) was determined in protein crude extracts of the Δ*hmgX* mutant, the wild type and the complemented strain *hmgX*c cultivated with or without tyrosine (Fig. 5). Without tyrosine, only basal Tat activity and no HmgA activity was detected in all strains. Remarkably, after addition of tyrosine a strong increase in Tat and HmgA activity occurred, which was always slightly stronger in the Δ*hmgX* mutant. Heat inactivated crude extracts showed neither Tat nor HmgA activity (data not shown).

**Figure 6:**
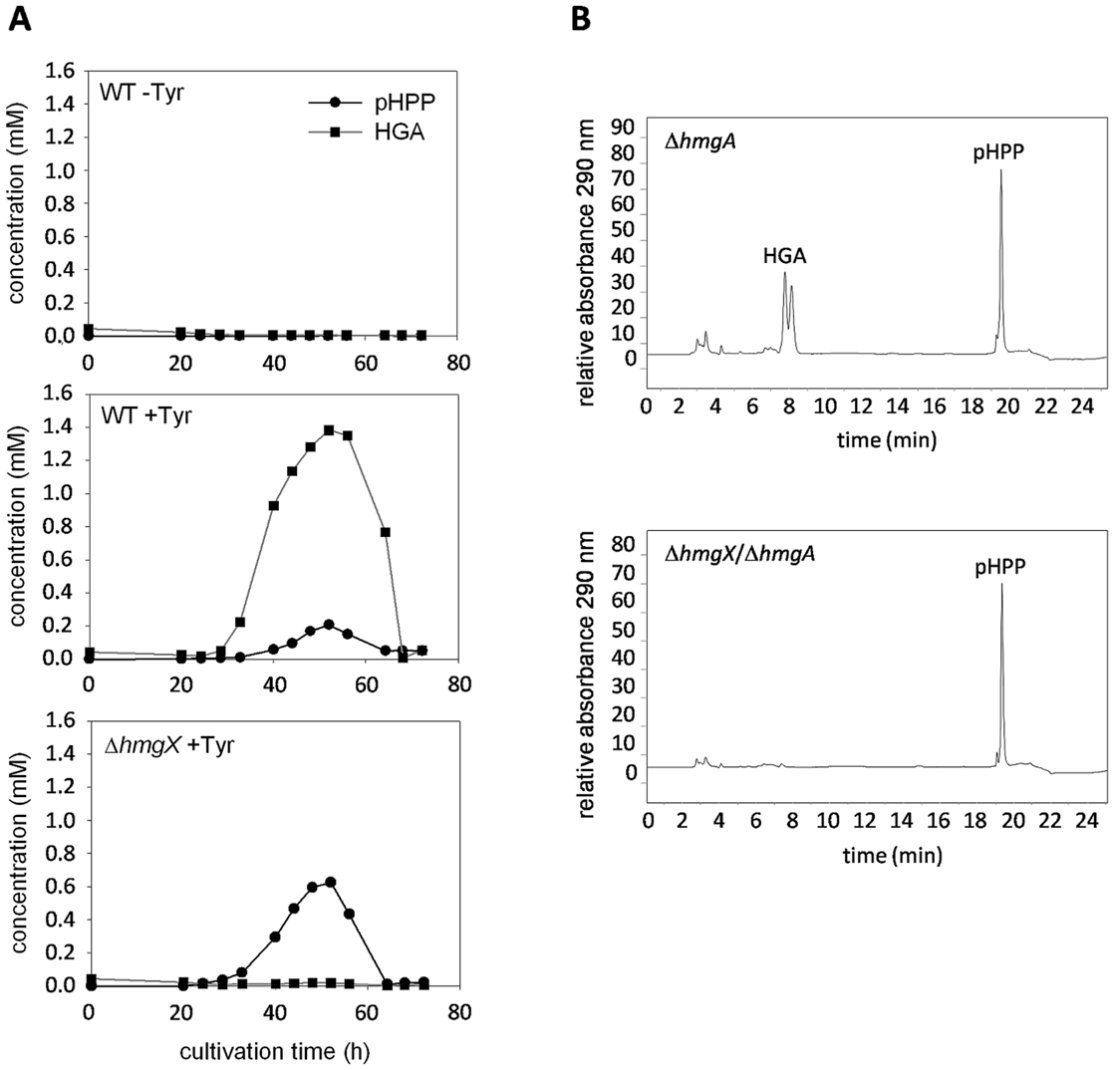
Formation of *p*-hydroxyphenylpyruvate and homogentisate. (A) The wild type (WT) and the Δ*hmgX* mutant were cultivated with (+) or without (-) addition of tyrosine. At the time points indicated, samples of the culture supernatant were taken and analyzed by HPLC to quantify formation of *p*-hydroxyphenylpyruvate (*p*HPP) and homogentisate (HGA). (B) Strains Δ*hmgA* and Δ*hmgX*/Δ*hmgA* were cultivated in presence of tyrosine. Conversion of *p*HPP to HGA by protein crude extracts was monitored by HPLC.

Due to the fact that HppD activity can not be determined in a spectrophotometric assay, the formation of the HppD substrate *p*HPP and the product HGA was monitored by HPLC analyses in culture supernatants of the wild type and the Δ*hmgX* mutant grown in the presence of tyrosine (Fig. 6A). In a control experiment in which the wild type was cultivated without addition of tyrosine, neither *p*HPP nor HGA were detected. In the wild-type culture supplemented with tyrosine, the concentration of *p*HPP only increased slightly. However, in the Δ*hmgX* culture the *p*HPP concentration significantly increased with a maximum at 56 h. HGA was only detected in wild-type but not in Δ*hmgX* cultures after addition of tyrosine.

To further analyze the role of HmgX, the conversion of *p*HPP to HGA was monitored by HPLC in tyrosine-induced protein crude extracts of strain Δ*hmgA* and a Δ*hmgX*/Δ*hmgA* double mutant (Fig. 6B; Fig. S6). Using the crude extract from Δ*hmgA*, *p*HPP was converted to HGA which accumulated due to the lack of HmgA activity. By contrast, in the crude extract from the double deletion strain Δ*hmgX*/Δ*hmgA* no HGA was detected, indicating that HmgX is essential to convert *p*HPP to HGA.

### Transcription of genes of the tyrosine degradation cluster during infection and analysis of the role of pyomelanin formation in pathogenicity

**Figure 7:**
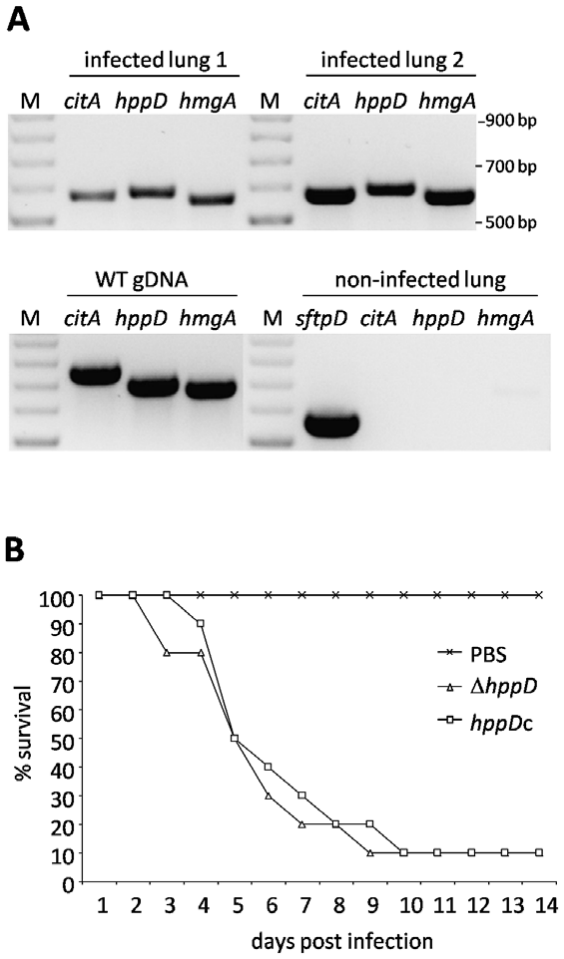
Role of tyrosine degradation and pyomelanin formation in pathogenicity. (A) Transcription of *hmgA* and *hppD* in lungs of infected mice. Genomic DNA (gDNA) from *A. fumigatus* wild type was used as control. Two lungs isolated from immunosuppressed mice, which were intranasally infected with *A. fumigatus* conidia, provided material for cDNA synthesis. A noninfected (PBS) lung was used as control. Transcription of fungal genes was analyzed by reverse transcription-PCR. Transcription of *A. fumigatus citA* (citrate synthase) was used as control and transcripts of murine surfactant protein D (*sftpD*) ensured the quality of mouse cDNA. M denotes a 100 bp DNA ladder. (B) Virulence of strain Δ*hppD* in a murine infection model for invasive aspergillosis. Survival of corticosteroid-treated mice after intranasal infection with the Δ*hppD* mutant and the corresponding complemented strain *hppD*c was monitored over a period of 14 days. Infections were performed with a group of 10 mice for each strain tested.

Finally, the potential role of tyrosine degradation and pyomelanin formation for pathogenicity of *A. fumigatus* was examined. First, transcription of *hppD* and *hmgA* was determined in lungs of immunocompromised mice intranasally infected with *A. fumigatus* wild-type conidia. The mice were sacrificed seven days post infection and cDNA was synthesized from isolated lung tissue. By reverse transcription PCR analysis, fungal mRNA steady-state levels of *hmgA* and *hppD* were compared to *A. fumigatus citA* transcripts (Fig. 7A) that served as control [34]. Additionally, cDNA synthesized from non-infected mice lungs was tested with the same oligonucleotides to ensure that the amplification products of *hppD* and *hmgA* did not derive from murine cDNA. As control for murine cDNA, the constitutively transcribed gene *sftpD*, encoding murine surfactant protein D, was used [35]. In the non-infected control lung, transcripts of *sftpD* were detected, whereas no amplification of the *A. fumigatus* specific genes *citA*, *hppD* and *hmgA* occurred. Similar mRNA steady-state levels for *hmgA*, *hppD* and *citA* were found in cDNA samples obtained from infected lungs. The ratios of *hmgA* and *hppD* compared to *citA* indicated the induction of the tyrosine degradation cluster *in vivo*. Therefore, tyrosine seems to be available in the lung and is metabolized to HGA by *A. fumigatus* during invasive growth.

Next, the virulence of the Δ*hppD* mutant, deficient for pyomelanin production due to deletion of the *p*-hydroxyphenylpyruvate dioxygenase encoding gene, was compared to the corresponding *hppD* reconstituted strain *hppD*c, that regained the full ability to degrade tyrosine and to produce pyomelanin. Both strains caused the same absolute mortality and similar survival kinetics after infection of corticosteroid treated mice (Fig. 7B). Due to immune suppression by cortisone acetate recruitment of neutrophils and monocytes to the site of infection still occurs. In accordance to the results obtained with the Δ*hppD* mutant, virulence studies with *hmgR* mutant strains revealed as well no differences in mortality (Fig. S7A). The presence of invasive mycelia in the lungs of leucopenic mice infected with Δ*hmgR* or *hmgR*c was confirmed by histopathology (Fig. S7B). The lungs of PBS-infected mice are shown as control. In the infected lungs, invasive hyphae are visible, surrounded by immune cells. The presence of the immune cells prevents rapid fungal dissemination and the mice die due to bronchopneumonia and not by hyphal growth *per se*.

Taken together, although genes responsible for tyrosine degradation and pyomelanin formation were expressed during infection, they are dispensable for virulence in corticosteroid-treated mice.

## Discussion

In *A. fumigatus*, tyrosine degradation occurs *via* HGA as the central intermediate. HGA either can be degraded enzymatically to acetoacetate and fumarate or can polymerize to the brownish pigment pyomelanin [13]. The products acetoacetate and fumarate can be used as carbon source. Additionally, tyrosine can be used as nitrogen source by transferring the amino residue of tyrosine *via* tyrosine aminotransferase activity to α-ketoglutarate resulting in the formation of glutamate that can be further used for synthesis of amino acids.

The genes involved in tyrosine catabolism are organized in a cluster within the genome of *A. fumigatus*. This cluster is also present in all other *Aspergillus* species for which full genome sequences are available, e.g., *A. clavatus*, *N. fischeri*, *A. niger, A. flavus*, *A. oryzae, A. nidulans*, and *A. terreus* (http://www.cadre-genomes.org.uk). Clustered organization of genes that are involved in the same metabolic process is not uncommon in filamentous fungi. For example, the genes for ethanol, proline, and nitrate catabolism are clustered, as are the genes for biosynthesis of secondary metabolites [36], [37], [38].

### HmgR is a transcriptional regulator of genes of the tyrosine degradation cluster

Tyrosine catabolism *via* HGA was investigated in detail in *A. nidulans* [19], [39], [40]. However, nothing was known about the transcriptional regulation of the relevant genes in fungi. In bacteria, regulation of tyrosine degradation has been studied for *Pseudomonas putida*. In this organism, the HGA degrading enzymes HmgA, MaiA and FahA are encoded by genes organized in a cluster. In *P. putida*, a gene designated *hmgR* is also part of the cluster. The corresponding gene product HmgR functions as repressor of genes for HGA degradation [41]. In *A. fumigatus*, the clustered organization of genes involved in tyrosine degradation as well as their concerted transcription in the presence of tyrosine imply the existence of a common regulator. Within the cluster, *hmgR* encodes a putative transcription factor with a Zn(II)2Cys6-DNA-binding domain, which is exclusively found in fungi [42]. This type of transcription factors often harbors activation- and dimerization domains, enabling them to bind to DNA as homo- or heterodimers and act as activators or repressors [43]. HmgR exhibits 40%identity to the transcriptional regulator Aro80p of *Saccharomyces cerevisiae*. Aro80p was shown to be involved in regulation of tyrosine degradation in yeast by binding to the *aro9* promoter and subsequent activation of *aro9* transcription in the presence of aromatic amino acids. Aro9p is the responsible enzyme for transamination of tyrosine to *p*HPP [44], [45]. Here, we could show that HmgR plays an important role in mediating tyrosine-induced transcription of all genes of the tyrosine degradation cluster. An HmgR-eGFP fusion protein was found to be localized in the nucleus, furthermore corroborating the hypothesis that HmgR functions as a transcriptional regulator. Consistently, in the Δ*hmgR* mutant transcription of the tyrosine degradation cluster genes was nearly completely abolished.

### HmgR and HmgX play key roles in tyrosine degradation and pyomelanin formation in *A. fumigatus*

As shown here, transcription of all genes was induced in the presence of tyrosine or phenylalanine, indicating that *hmgR* and *hmgX* are involved in tyrosine degradation. Furthermore, we demonstrated that the tyrosine degradation cluster only consists of six genes, as exclusively transcription of these genes and not the adjacent genes (AFUA_2G04270 and AFUA_2G04190) was influenced by the presence of tyrosine. In contrast to the wild type and complemented strains, neither Δ*hmgX* nor Δ*hmgR* were able to use tyrosine as sole carbon or nitrogen source, indicating that these genes are essential for tyrosine catabolism. The inability to catabolize tyrosine is also illustrated by the finding that tyrosine crystals were not degraded by the mutants, but by the wild type. A similar finding was previously described for the bacterium *Sinorhizobium meliloti* [46]. Furthermore, deletion of either *hmgR* or *hmgX* significantly impaired pyomelanin formation in tyrosine-containing medium. However, only Δ*hmgX* completely failed to produce the pigment, as it was previously shown for the Δ*hppD* mutant [13]. Remarkably, inhibition of pyomelanin production was less pronounced in the Δ*hmgR* mutant compared to Δ*hmgX* and residual pyomelanin was formed in Δ*hmgR*. This is consistent with the finding that some residual transcription still occurred in the Δ*hmgR* mutant, implying that HmgR is the main, but not the only transcription factor involved in tyrosine catabolism.

Interestingly, although HppD is generally conserved between a wide range of organisms, HppD in ascomycetes which also harbor an HmgX homolog is distinguished by a 13 aa sequence at the N-terminal region to organisms without an HmgX homolog. Thus, this sequence might represent a protein interaction domain, important for defined HppD activity depending on HmgX. This is supported by the complete lack of HGA formation in a Δ*hmgX*/Δ*hmgA* double mutant. In this mutant, any HGA that is produced should accumulate. However, no HGA was detected by HPLC in protein crude extracts of strain Δ*hmgX*/Δ*hmgA* revealing that the conversion of *p*HPP to HGA by enzymatic activity of HppD depends on the presence of HmgX. In the control strain Δ*hmgA*, HGA accumulated as expected. These data suggest that HmgX might function as an accessory factor mediating specific activity of HppD by a yet unidentified mechanism.

### Pyomelanin formation is dispensable for virulence in a murine infection model for pulmonary aspergillosis

*A. fumigatus* secretes a wide variety of proteases during invasive growth in lung tissue [47], [48], [49]. Thus, phenylalanine and tyrosine are likely available as substrates for pyomelanin synthesis during infection. Supporting this hypothesis, we observed that transcription of the tyrosine degradation genes was induced in lungs of infected mice. Pyomelanin protects the fungus against ROI [13] and, additionally, pyomelanin synthesis was found to be increased by cell wall stress, implying a function of pyomelanin in rescuing cell wall integrity [50]. However, neither the ability of *A. fumigatus* to detoxify host-produced ROI nor the sensing of impaired cell wall integrity contributes to virulence [51], [52], [53]. In accordance with these findings, we clearly showed that despite induction of transcription of genes involved in tyrosine degradation during infection, tyrosine catabolism and pyomelanin formation do not play an essential role in *A. fumigatus* induced mortality at least in a murine infection model for pulmonary aspergillosis. Nevertheless, a possible impact on fungal pathogenesis remains unclear. It cannot be ruled out that in a different infection model and in other host organisms the ability to degrade tyrosine and to synthesize pyomelanin might contribute to virulence and have an impact on pathogenicity of *A. fumigatus*.

## Supporting Information

Figure S1 **Alignment of HppD protein sequences.** Protein sequences of HppD of *Homo sapiens*, *Caenorhabditis elegans*, and *Dictyostelium discoideum* were aligned to HppD sequences from the ascomycetes *A. fumigatus*, *Coccidioides immitis*, *Neurospora crassa* und *Magnaporthe grisea*. "*" depicts identical aa in that column in all sequences in the alignment; ":" indicates conserved substitutions in the respective column; "." means that semi-conserved substitutions are observed. The variable region in the N-terminus is boxed.
(TIF)

Figure S2 **Northern blot analysis of genes adjacent to the tyrosine degradation cluster.** To determine cluster borders *A. fumigatus* wild type was cultivated for 12 h in AMM with (+) or without (-) L-tyrosine after a pre-cultivation for 16 h. The mRNA steady state levels were monitored for AFUA_2G04190 and AFUA_2G04270 by Northern blot analysis.
(TIF)

Figure S3 **Generation of** ***hmgX*** **disruption and complemented strains.** Schematic drawing of the genomic situation in the wild type (A) and *ΔhmgX* (B) as well as the plasmid pCR2.1 *hmgX*c (C) that was used for generation of the complemented strain *hmgX*c. For Southern blot analysis (D) chromosomal DNA was digested with restriction endonuclease *Nco*I yielding a 5.0 kb band for the wild type (WT). This band disappeared in *ΔhmgX* (ΔX). Instead a 2.7 kb band was visible indicating the insertion of the pyrithiamine resistance cassette and therefore disruption of the *hmgX* gene. The complemented strain *hmgX*c (Xc) showed the same 2.7 kb band and an additional one representing an ectopic integration of *hmgX* in the *ΔhmgX* mutant. The probe used for Southern blot hybridizes with the *hmgX* gene and the 3′ intergenic region.
(TIF)

Figure S4 **Generation of** ***hmgR*** **null mutant and complemented strains.** Schematic representation of the chromosomal *hmgR* locus in the wild type (A) and the *hmgR* deletion mutant (B) is shown. Generation of the complemented strain *hmgR*c was performed with plasmid pUCGH-*hmgR* (C), which harbors an *hmgR*-*egfp* fusion gene under the control of the native *hmgR* promoter. For Southern blot analysis (D) genomic DNA of the wild type (WT), *ΔhmgR* (ΔR) and the reconstituted strain *hmgR*c (Rc) was digested with *Eco*NI. In the *hmgR* deletion strain, the 5.1 kb wild-type signal was absent and a 9.5 kb DNA fragment appeared, indicating the replacement of *hmgR* with the *ptrA* sequence. In the *hmgR*c strain, two additional bands appeared, indicating double integration of plasmid pUCGH-*hmgR*. Restriction endonuclease sites of *Eco*NI and the position to which the probe for Southern blot analysis hybridizes, are indicated.
(TIF)

Figure S5 **Generation of strain** ***hmgX-egfp*** **.** Schematic drawing of the genomic situation in the wild type (A) and the plasmid pUCGH *hmgX*p-*hmgX* (B) that was used for generation of an *hmgX*-*egfp* fusion gene under control of the native *hmgX* promoter region. For Southern blot analysis (C) genomic DNA of the wild type (WT) and strain *hmgX*p-*hmgX*-*egfp* (*hmgX-egfp*) was digested with restriction endonuclease *Sal*I. The resulting 10.3 kb wild-type band was also present in strain *hmgX*p-*hmgX*-*egfp*. In this case,four additional signals were detectable representing four ectopic integrations of the plasmid. The probe used for Southern blot analysis binds to the *hmgX* gene.
(TIF)

Figure S6 **Generation of strain Δ** ***hmgX/*** **Δ** ***hmgA*** **.** Schematic drawing of the genomic situation in the wild type (A) and Δ*hmgX/*Δ*hmgA* (B). For Southern blot analysis (C) chromosomal DNA was digested with restriction endonucleases *Acc65*I and *Not*I. The 3.3 kb band characteristic for the wild type (WT) disappeared in the double mutant Δ*hmgX/*Δ*hmgA* (ΔXΔA) where instead a 6.0 kb signal was detected. This indicates that both *hmgX* and *hmgA* were partially replaced by the pyrithiamine resistance cassette. The probe used for Southern blot binds to the *hppD* gene.
(TIF)

Figure S7 **Virulence of strain Δ** ***hmgR*** **in a murine infection model.** (A) Survival of leucopenic CD-1 mice after infection with strains Δ*hmgR* and *hmgR*c. Infections were performed with a group of 10 mice for each tested strain. (B) Histopathology of representative sections of lungs 4 days post infection, using Periodic acid-Schiff (PAS, hyphae stain pink). The presence of invasive mycelia was confirmed in lungs of mice infected with Δ*hmgR* or *hmgR*c. The lung section of a PBS-infected mice is shown as control. Different sections of lungs of infected mice are shown, monitoring slight variations at the sites of infection within the same lung. However, no obvious qualitative differences can be detected between infections with Δ*hmgR* and *hmgR*c strains. In both, Δ*hmgR* and *hmgR*c infected lungs invasive hyphae are visible, surrounded by immune cells.
(TIF)

Table S1 ***A. fumigatus*** **strains used in this study.**
(DOC)

Table S2 **Oligonucleotides used in this study.**
(DOC)
