# Supplementary figures and images for: Pyomelanin Formation in Aspergillus fumigatus Requires HmgX and the Transcriptional Activator HmgR but Is Dispensable for Virulence
Source: PLoS One. 2011 Oct 27;6(10):e26604. doi: 10.1371/journal.pone.0026604 (PMC3203155; doi:10.1371/journal.pone.0026604)

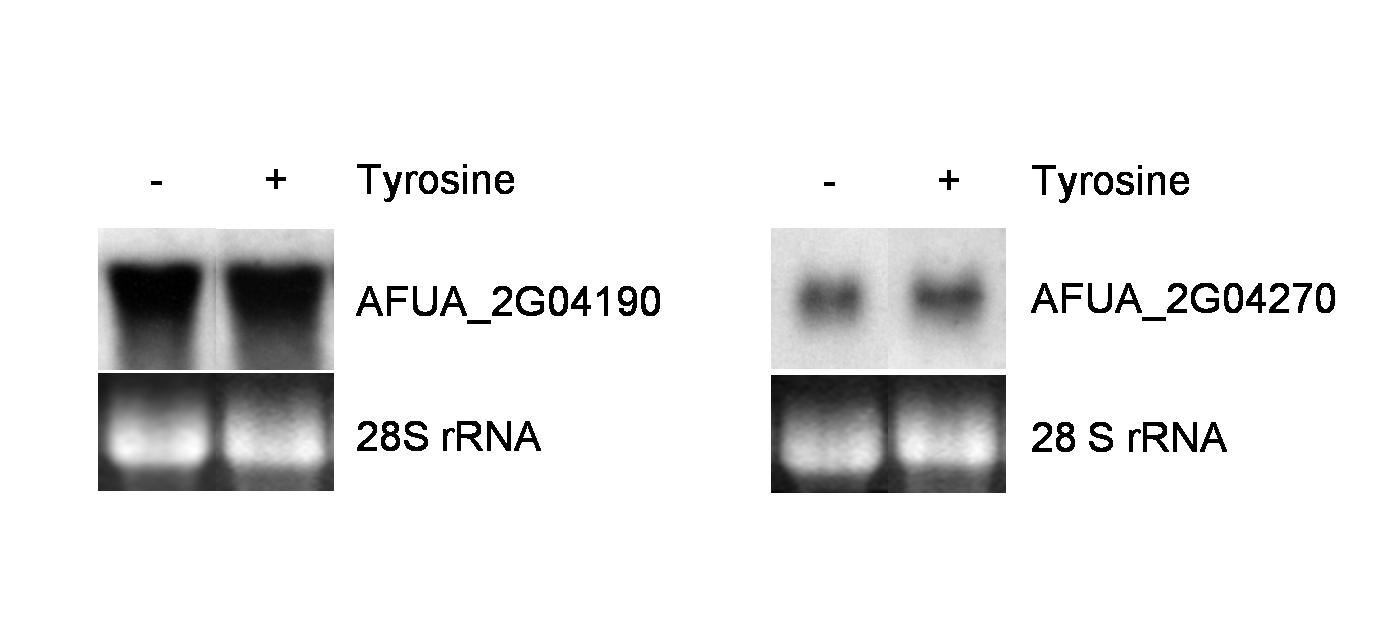

Supplement: Figure S2 — Northern blot analysis of genes adjacent to the tyrosine degradation cluster. To determine cluster borders A. fumigatus wild type was cultivated for 12 h in AMM with (+) or without (-) L-tyrosine after a pre-cultivation for 16 h. The mRNA steady state levels were monitored for AFUA_2G04190 and AFUA_2G04270 by Northern blot analysis. (TIF) [file pone.0026604.s002.tif]

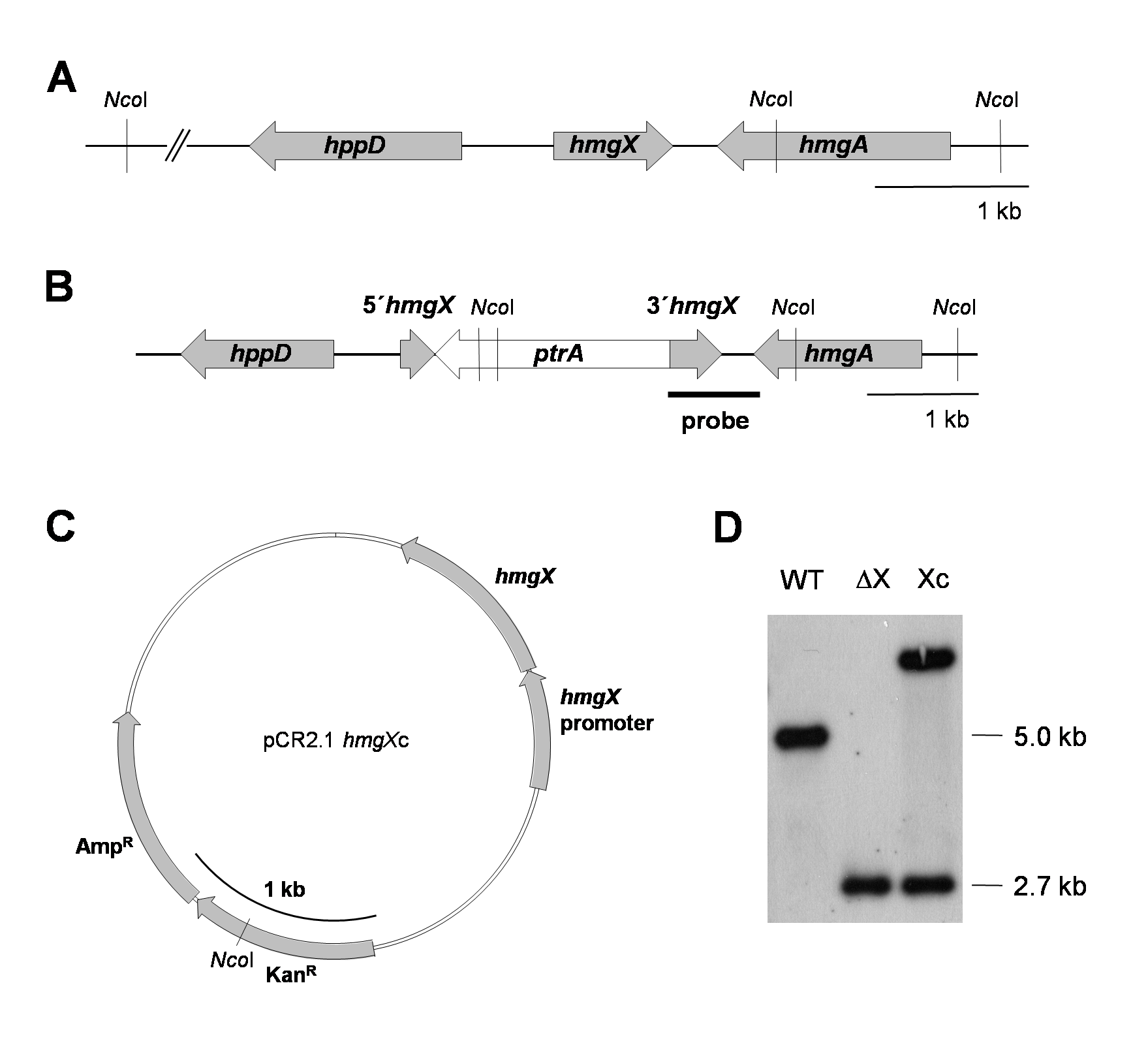

Supplement: Figure S3 — Generation of hmgX disruption and complemented strains. Schematic drawing of the genomic situation in the wild type (A) and ΔhmgX (B) as well as the plasmid pCR2.1 hmgXc (C) that was used for generation of the complemented strain hmgXc. For Southern blot analysis (D) chromosomal DNA was digested with restriction endonuclease NcoI yielding a 5.0 kb band for the wild type (WT). This band disappeared in ΔhmgX (ΔX). Instead a 2.7 kb band was visible indicating the insertion of the pyrithiamine resistance cassette and therefore disruption of the hmgX gene. The complemented strain hmgXc (Xc) showed the same 2.7 kb band and an additional one representing an ectopic integration of hmgX in the ΔhmgX mutant. The probe used for Southern blot hybridizes with the hmgX gene and the 3′ intergenic region. (TIF) [file pone.0026604.s003.tif]

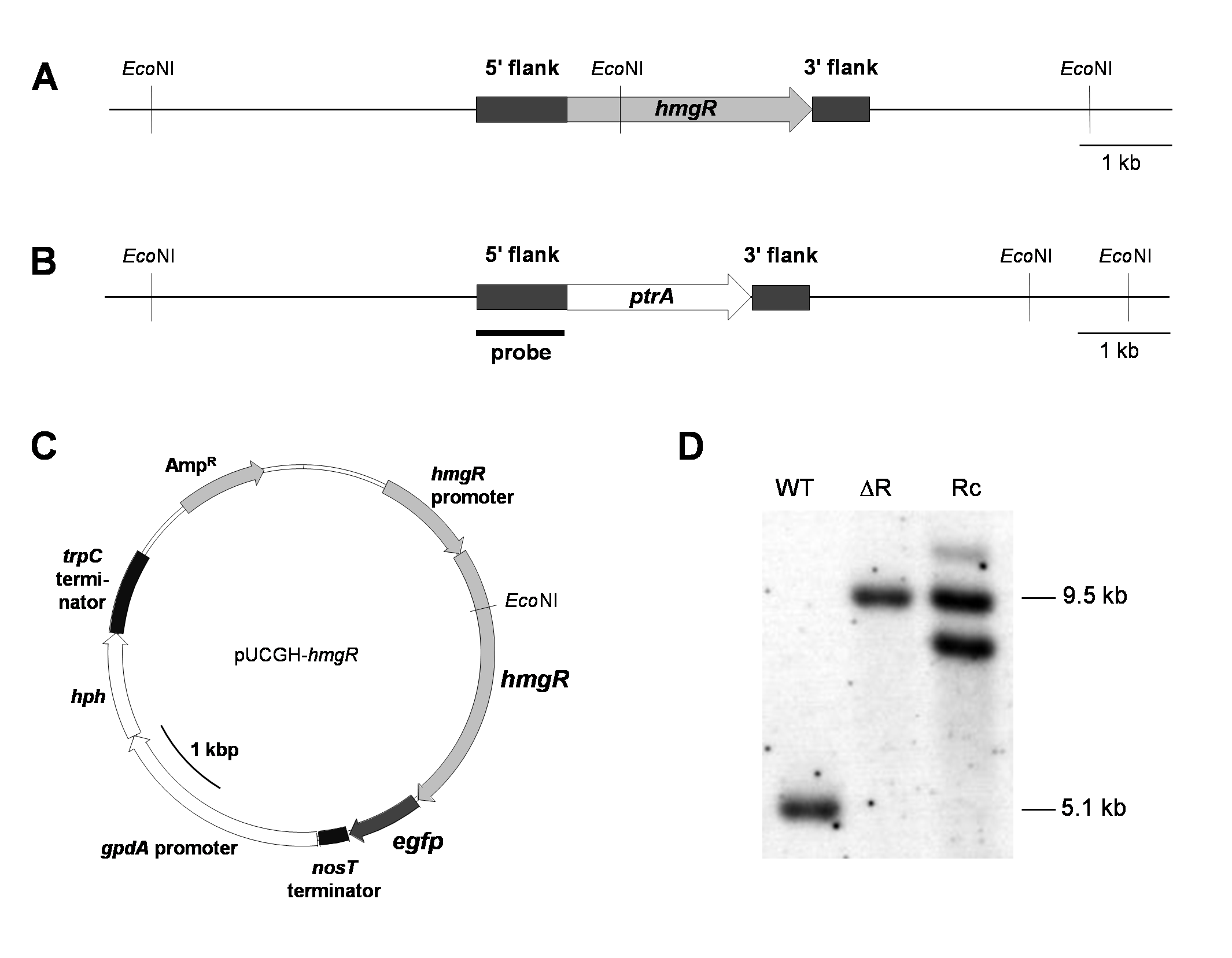

Supplement: Figure S4 — Generation of hmgR null mutant and complemented strains. Schematic representation of the chromosomal hmgR locus in the wild type (A) and the hmgR deletion mutant (B) is shown. Generation of the complemented strain hmgRc was performed with plasmid pUCGH-hmgR (C), which harbors an hmgR-egfp fusion gene under the control of the native hmgR promoter. For Southern blot analysis (D) genomic DNA of the wild type (WT), ΔhmgR (ΔR) and the reconstituted strain hmgRc (Rc) was digested with EcoNI. In the hmgR deletion strain, the 5.1 kb wild-type signal was absent and a 9.5 kb DNA fragment appeared, indicating the replacement of hmgR with the ptrA sequence. In the hmgRc strain, two additional bands appeared, indicating double integration of plasmid pUCGH-hmgR. Restriction endonuclease sites of EcoNI and the position to which the probe for Southern blot analysis hybridizes, are indicated. (TIF) [file pone.0026604.s004.tif]

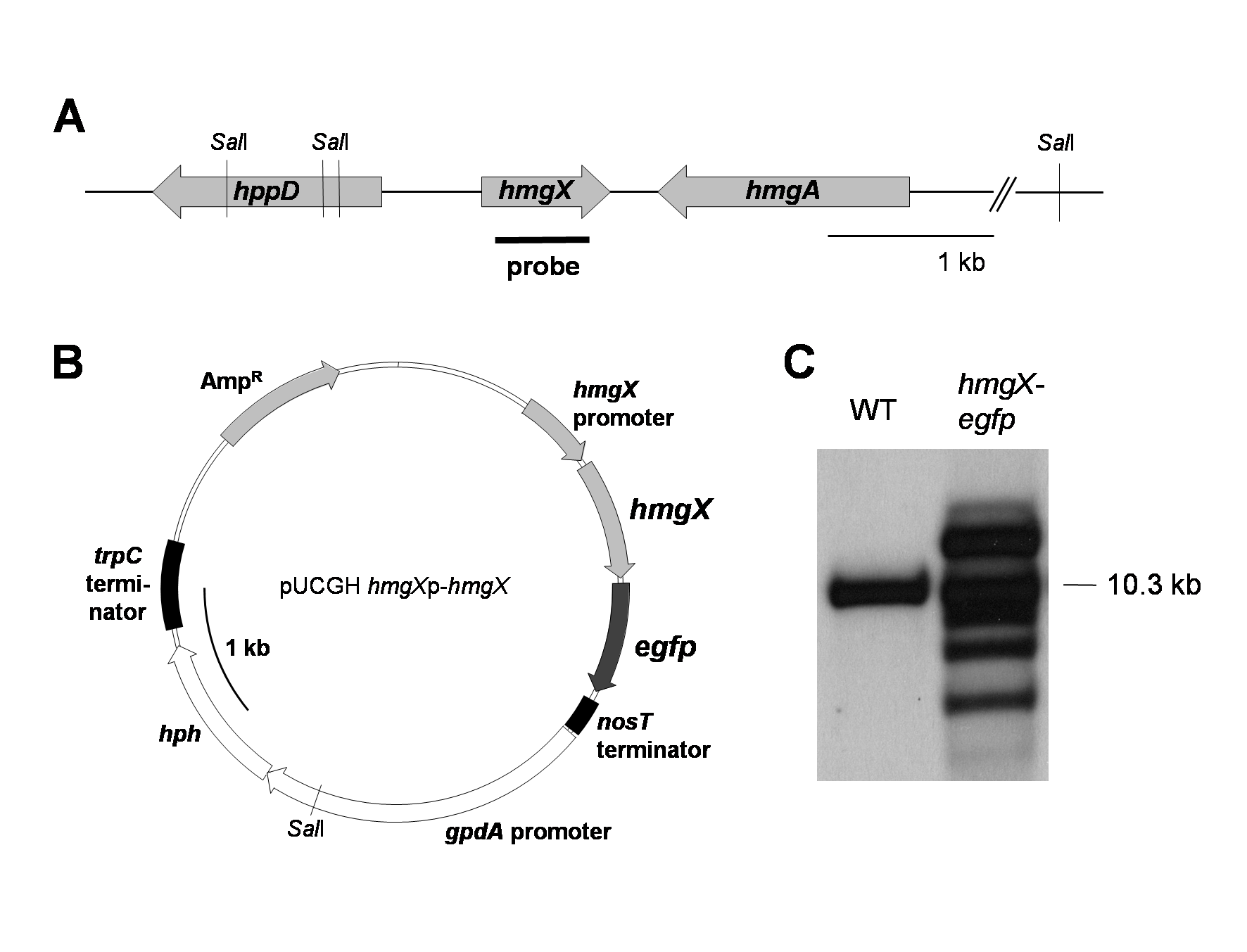

Supplement: Figure S5 — Generation of strain hmgX-egfp . Schematic drawing of the genomic situation in the wild type (A) and the plasmid pUCGH hmgXp-hmgX (B) that was used for generation of an hmgX-egfp fusion gene under control of the native hmgX promoter region. For Southern blot analysis (C) genomic DNA of the wild type (WT) and strain hmgXp-hmgX-egfp (hmgX-egfp) was digested with restriction endonuclease SalI. The resulting 10.3 kb wild-type band was also present in strain hmgXp-hmgX-egfp. In this case,four additional signals were detectable representing four ectopic integrations of the plasmid. The probe used for Southern blot analysis binds to the hmgX gene. (TIF) [file pone.0026604.s005.tif]

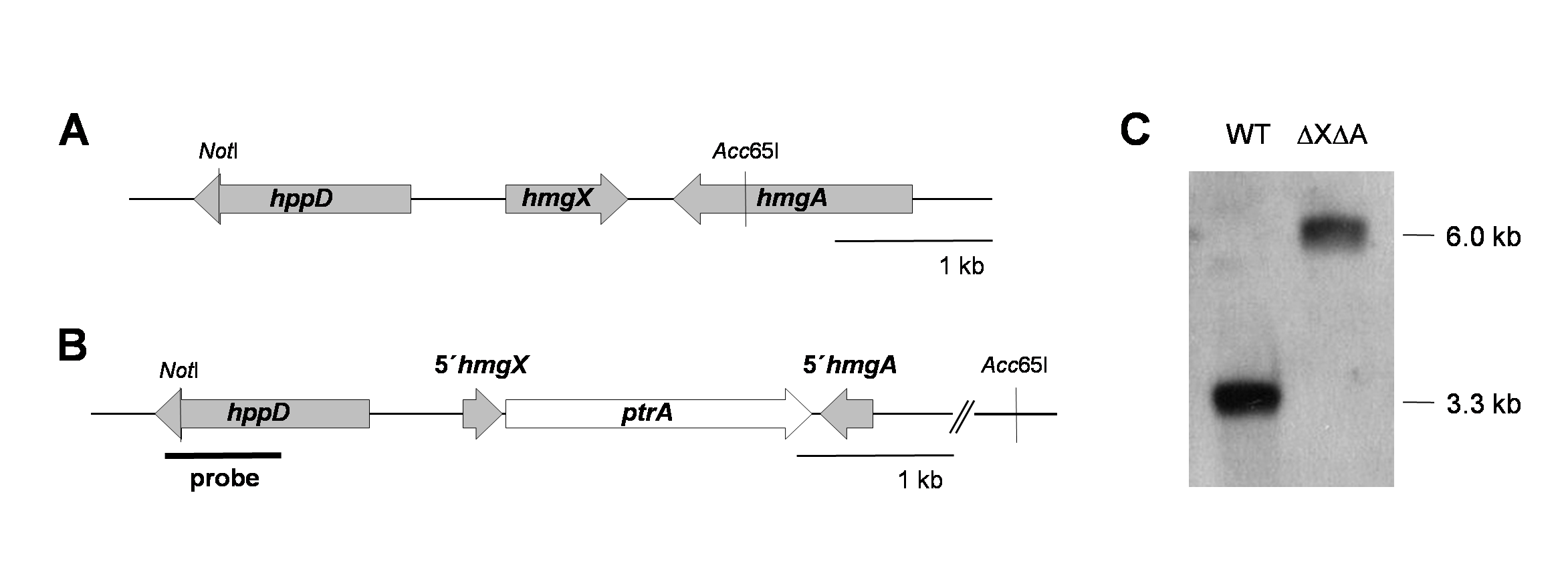

Supplement: Figure S6 — Generation of strain Δ hmgX/ Δ hmgA . Schematic drawing of the genomic situation in the wild type (A) and ΔhmgX/ΔhmgA (B). For Southern blot analysis (C) chromosomal DNA was digested with restriction endonucleases Acc65I and NotI. The 3.3 kb band characteristic for the wild type (WT) disappeared in the double mutant ΔhmgX/ΔhmgA (ΔXΔA) where instead a 6.0 kb signal was detected. This indicates that both hmgX and hmgA were partially replaced by the pyrithiamine resistance cassette. The probe used for Southern blot binds to the hppD gene. (TIF) [file pone.0026604.s006.tif]

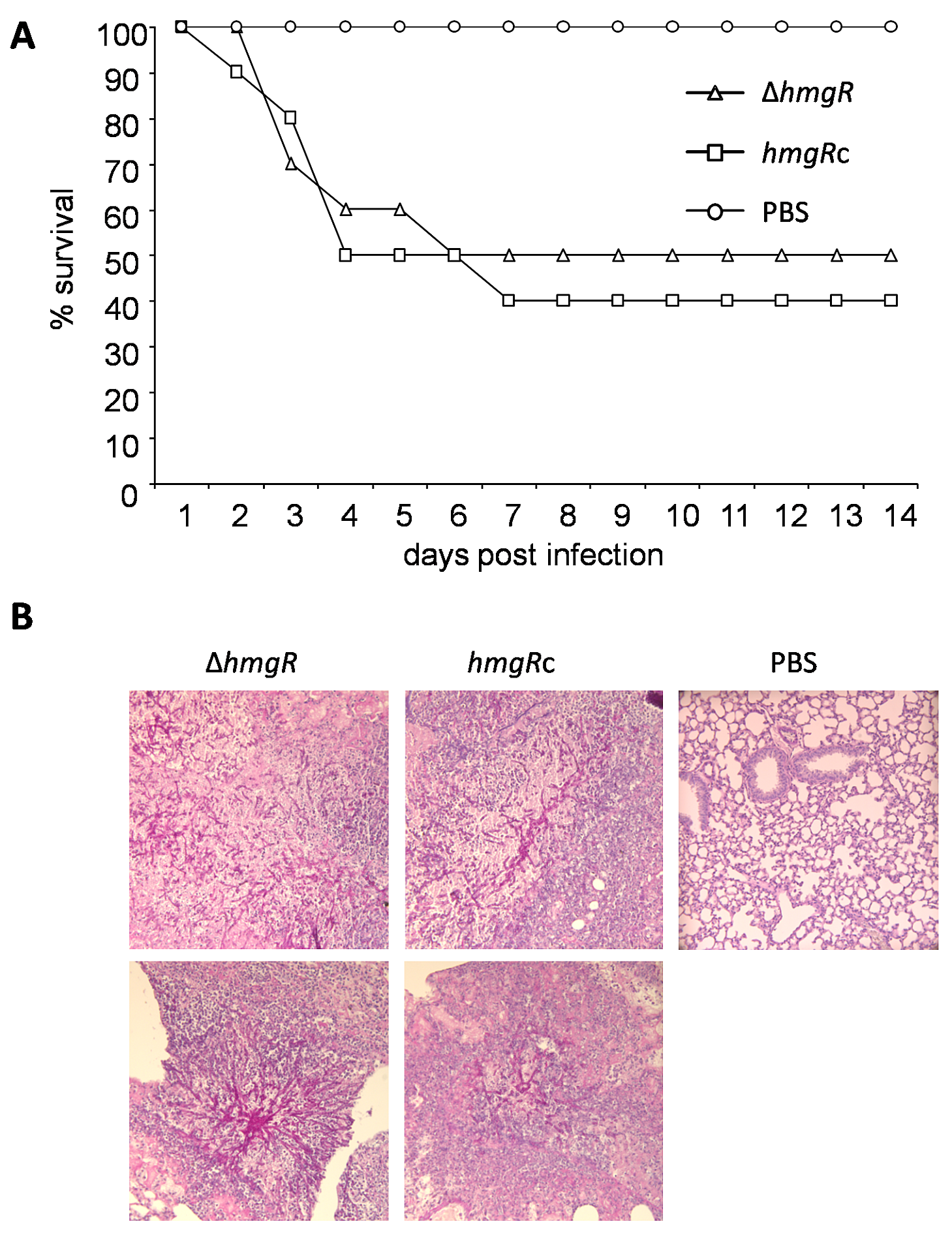

Supplement: Figure S7 — Virulence of strain Δ hmgR in a murine infection model. (A) Survival of leucopenic CD-1 mice after infection with strains ΔhmgR and hmgRc. Infections were performed with a group of 10 mice for each tested strain. (B) Histopathology of representative sections of lungs 4 days post infection, using Periodic acid-Schiff (PAS, hyphae stain pink). The presence of invasive mycelia was confirmed in lungs of mice infected with ΔhmgR or hmgRc. The lung section of a PBS-infected mice is shown as control. Different sections of lungs of infected mice are shown, monitoring slight variations at the sites of infection within the same lung. However, no obvious qualitative differences can be detected between infections with ΔhmgR and hmgRc strains. In both, ΔhmgR and hmgRc infected lungs invasive hyphae are visible, surrounded by immune cells. (TIF) [file pone.0026604.s007.tif]
