# Supplementary material for: Pyomelanin Formation in Aspergillus fumigatus Requires HmgX and the Transcriptional Activator HmgR but Is Dispensable for Virulence
Source: PLoS One. 2011 Oct 27;6(10):e26604. doi: 10.1371/journal.pone.0026604 (PMC3203155; doi:10.1371/journal.pone.0026604)
Supplement: Table S1 — A. fumigatus strains used in this study. (DOC) [file pone.0026604.s008.doc]

**Table S1. *A. fumigatus* strains used in this study**

| **Strain** | **Description** | **Reference** |
| --- | --- | --- |
| CEA10 | wild type | CBS 144-89 |
| Δ*akuB* | *akuB^KU80^::pyrG*; pyrG^+^, Δ*akuB* | [1] |
| Δ*hmgX* | derived from Δ*akuB*; *hmgX::ptrA*; PT^R^, Δ*hmgX* | this study |
| *hmgX*c | derived from Δ*hmgX*; *hmgX^+^* | this study |
| Δ*hppD* | derived from Δ*akuB*; *hppD::ptrA*; PT^R^, Δ*hppD* | [2] |
| *hppD*c | derived from Δ*hppD*; *hppD^+^* | [2] |
| *hmgX*p*-hmgX-egfp* | derived from CEA10; contains *hmgX*-*egfp-*fusion construct under control of the *hmgX*-promoter; Hyg^R^ | this study |
| Δ*hmgR* | derived from Δ*akuB*; *hmgR::ptrA*; PT^R^, Δ*hmgR* | this study |
| *hmgR*p*-hmgR-egfp = hmgR*c | derived from Δ*hmgR*; contains *hmgR*-*egfp-*fusion construct under control of the *hmgR*-promoter; Hyg^R^ | this study |
| Δ*hmgA* | derived from Δ*akuB*; *hmgA::ptrA*; PT^R^, Δ*hmgA* | [2] |
| Δ*hmgX*/Δ*hmgA* | derived from Δ*akuB*; *hmgX-hmgA::ptrA*; PT^R^, Δ*hmgX,* Δ*hmgA* | this study |

PT^R^: Pyrithiamine-resistance; Hyg^R^: Hygromycin-resistance

**References for supplemental table S1**

1. da Silva Ferreira ME, Kress MR, Savoldi M, Goldman MH, Hartl A, et al. (2006) The *akuB*(KU80) mutant deficient for nonhomologous end joining is a powerful tool for analyzing pathogenicity in *Aspergillus fumigatus*. Eukaryot Cell 5: 207-211.

2. Schmaler-Ripcke J, Sugareva V, Gebhardt P, Winkler R, Kniemeyer O, et al. (2009) Production of pyomelanin, a second type of melanin, via the tyrosine degradation pathway in *Aspergillus fumigatus*. Appl Environ Microbiol 75: 493-503.
