# Supplementary material for: Pyomelanin Formation in Aspergillus fumigatus Requires HmgX and the Transcriptional Activator HmgR but Is Dispensable for Virulence
Source: PLoS One. 2011 Oct 27;6(10):e26604. doi: 10.1371/journal.pone.0026604 (PMC3203155; doi:10.1371/journal.pone.0026604)
Supplement: Table S2 — Oligonucleotides used in this study. (DOC) [file pone.0026604.s009.doc]

**Table S2. Oligonucleotides used in this study**

| **Oligonucleotide** | **Sequence (5' - 3')** | **target gene** |
| --- | --- | --- |
| AfFahAcode_for | GTTTCGCGGTCCTGAGAACG | *fahA* |
| AfFahAcode_rev | GTGGTGTGCCAGCATCTGAG | *fahA* |
| AfHmgAcode_down | ACCAGACTGGCTTCGACTCC | *hmgA* |
| AfHmgAcode_up | ACCTGCTGGAAGGGTGACAC | *hmgA* |
| AfHmgXcode_for | CTTCGACGCCGAATCATCCC | *hmgX* |
| AfHmgXcode_rev | TCCCTACTCGCCATCTCCTCC | *hmgX* |
| AfHppDcode2_for | TGCGCAACGGCGACATCAC | *hppD* |
| AfHppDcode2_rev | TGGCGGGTTCGTTGATGGG | *hppD* |
| AfMaiAcode_for | TCCTCCTGCTCAGCCAGAC | *maiA* |
| AfMaiAcode_rev | CAATGAGCCCTCTTCACCG | *maiA* |
| AfPyoTFcode-for | GCGATGCTTTCGTCGCCTAC | *hmgR* |
| AfPyoTFcode-rev | GCAAGCTGCGTGACCTTCTC | *hmgR* |
| HmgX-Acc65Ifor | GGTACCGAAGTCAAGTGAGAAGAGATG | *hmgX* |
| HmgX-BamHIrev | GGATCCAATAACCCCCTCAACTTCC | *hmgX* |
| hmgX3rev | CTCATGTGTCGTCGCCATTTGC | *hmgX* |
| hmgX5for | GTCTGTTGAGCTTGAACCATTG | *hmgX* |
| hmgX-ptrA5rev | GGCCTGAGTGGCCATCGAATTCCCTGATGGCGGATGTGATGG | *hmgX* |
| hmgA5rev | CAAGATGATCCAGAGCGACC | *hmgA* |
| hmgA-ptrA5for | GAGGCCATCTAGGCCATCAAGCGTGCGTCTCCGTAGTCATGC | *hmgA* |
| Tf_Tyr_up | CCTGATCGTTTACATGCGGAGG | *hmgR* |
| Tf_Tyr_down | GTTGAGAGTCGAGAGGGTCAAG | *hmgR* |
| Tf_Tyr_SfiI_up | AGGCCTGAGTGGCCAGTGTGACCAATCACATCATGG | *hmgR* |
| Tf_Tyr_SfiI_down | AGGCCATCTAGGCCAGTCGTTTACTACTGAGATTCTTG | *hmgR* |
| PyoTf-BamHIrev | GGATCCCCATAGGAAATTCAAGCCGC | *hmgR* |
| PyoTf5'-Acc65I | GGTACCTCCTGATCGTTTACATGCGG | *hmgR* |
| CHY2_for | TCAAGCAGGAGCCAAGTCGG | AFUA_2G04190 |
| CHY2_rev | GACGAGGCACCAACAACTTCAC | AFUA_2G04190 |
| TIM17_for | CGAGAGATCCCTGTCCCTGG | AFUA_2G04270 |
| TIM17_rev | GTTGTCCGCCATCATCCTCTGG | AFUA_2G04270 |
| ptrAforII | GAATTCGATGGCCACTCAGGCC | *ptrA* |
| ptrArevII | GCTTGATGGCCTAGATGGCCTC | *ptrA* |
